# Objective assessment of the association between frailty and sedentary behavior in older adults: a cross-sectional study

**DOI:** 10.1186/s11556-023-00324-5

**Published:** 2023-08-07

**Authors:** Wen-Ning Chang, Pei-Lin Tzeng, Wei-Jia Huang, Yu-Hung Lin, Kun-Pei Lin, Chiung-Jung Wen, Yi-Chun Chou, Yung Liao, Ming-Chun Hsueh, Ding-Cheng Chan

**Affiliations:** 1 Department of Geriatrics and Gerontology, National Taiwan University Hospital, No. 7, Chungshan S. Rd, Taipei, 100225 Taiwan; 2 Department of Health Promotion and Health Education, National Taiwan Normal University, No 162, Section 1, Heping E. Rd, Taipei, 106209 Taiwan; 3 National Taiwan University Hospital, Bei-Hu Branch, No. 87 Neijiang Street, Taipei, 108206 Taiwan; 4 Department of Internal Medicine, National Taiwan University Hospital, No. 7, Chungshan S. Rd, Taipei, 100225 Taiwan; 5 Department of Family Medicine, College of Medicine, National Taiwan University, No. 7, Chungshan S. Rd, Taipei, 100225 Taiwan; 6 Graduate Institute of Sport, Leisure and Hospitality Management, National Taiwan Normal University, No. 162, Section 1, Heping E. Rd, Taipei, 106209 Taiwan; 7 Graduate Institute of Sport Pedagogy, University of Taipei, No. 101, Sec. 2, Zhongcheng Rd, Taipei, 111036 Taiwan

**Keywords:** Older adults, Frailty, Sedentary behavior, Accelerometer

## Abstract

**Background:**

Given the inconsistent findings of the association between frailty and sedentary behavior in older adults, this cross-sectional study investigated the aforementioned association using four different frailty criteria and two sedentary behavior indices in older adults.

**Methods:**

Data from older adults (age ≥ 65 y) who participated in health examinations or attended outpatient integrated clinics at a medical center in Taipei, Taiwan, were collected. Frailty was measured using the modified Fried Frailty Phenotype (mFFP), Clinical Frailty Scale in Chinese Translation (CFS-C), Study of Osteoporotic Fractures (SOF) index, and Clinical Frailty-Deficit Count (CF-DC) index; sedentary behavior was assessed with a waist-worn accelerometer. Adjusted linear regression ascertained the association between frailty and both sedentary behavior outcomes.

**Results:**

Among the 214 participants (mean age 80.82 ± 7.14 y), 116 were women. The average total sedentary time and number of sedentary bouts were 609.74 ± 79.29 min and 5.51 ± 2.09 times per day, respectively. Frail participants had a longer total sedentary time (odds ratio [OR]: 30.13, *P* = .01 and 39.43, *P* < .001) and more sedentary bouts (OR: 3.50 and 5.86, both *P* < .001) on mFFP and CFS-C assessments, respectively. The SOF index revealed more sedentary bouts among frail than in robust participants (OR: 2.06, *P* = .009), without a significant difference in the total sedentary time. Frail participants defined by the CF-DC index were more likely to have frequent sedentary bouts (OR: 2.03, *P* = .016), but did not have a longer total sedentary time.

**Conclusions:**

Regardless of the frailty criteria adopted, frailty was positively associated with the number of sedentary bouts per day in older adults. A significant correlation between frailty and total sedentary time was detected only with mFFP and CFS-C indices. Further research may target decreasing the sedentary bouts in older adults as a strategy to improve frailty.

## Background

Frailty, which is characterized as a clinical state wherein individuals are more vulnerable to stressor exposure [1], is a common health issue among older adults. However, there is a lack of consensus with regard to the definition of frailty worldwide. Among the several operational criteria for assessing frailty risk, two theories are most widely accepted: one theory involves the definition of frailty based on a phenotypic model, such as the Fried Frailty Phenotype (FFP) and the Study of Osteoporotic Fractures (SOF) index, that mainly focuses on the physical condition [2, 3]; another theory defines frailty on the basis of a deficit-accumulation model, such as the Frailty Index (FI) and the Clinical Frailty Scale (CFS), which include multiple domains [4, 5]. Depending on the diagnostic criteria adopted, the prevalence of frailty varies between 4.0% and 59.1% in community-dwelling older adults [6]. In Taiwan, the prevalence of frailty in the older population ranges from 4.9% to 42%, with varied associations that depend on the place of residence and measurement criteria [7–9]. Frailty is correlated with negative health outcomes, such as physical limitations, falls, fractures, hospitalization, and death [10]. The increasing demand from the frail older population for healthcare services increasingly constitutes a significant socioeconomic burden [11], and this issue deserves attention.

Sedentary behavior has emerged as a new risk factor for health [12] and is defined as any waking activity that requires energy expenditure of less than or equal to 1.5 basal metabolic equivalents with a sitting or reclining posture, such as television viewing, reading, and computer use [13]. Sedentary behavior is measured using self-reported questionnaires or accelerometers [14], which are objective assessment tools that can provide information on the wearer’s detailed patterns of daily physical activity. Several studies have demonstrated that older people tend to spend most of their time awake performing sedentary activities [15]. In Taiwanese older adults, the self-reported and objectively measured total sedentary time per day was 4.72 [16] and 10.1 h [17] respectively, which was similar to the global average (range, 5.3–9.4 h) [15]. Furthermore, strong evidence exists on the relationship between sedentary behavior and all-cause mortality, cardiovascular disease, and metabolic syndrome [12].

Investigations of the association between frailty and sedentary behavior in older adults are increasingly being conducted [18]. Nevertheless, the correlation of frailty with sedentary behavior in previous studies has been inconsistent [18, 19]. Most analyses found that greater sedentary behavior time was correlated with higher frailty [20–26] when FFP was applied mainly, but two still showed no significant association between sedentary behavior and frailty [27, 28]. The relationship between frailty and sedentary bouts was also investigated in three studies, two of which indicated that a higher number of sedentary bouts was associated with higher frailty [26, 29], while another study found that more time in sedentary bouts was non-significantly associated with frailty [27]. These discrepancies might result from participants' characteristics, adjusted variables, different measurements of sedentary behavior, and the heterogeneity of frailty assessments. The conclusion regarding the correlation between sedentary behavior and frailty may be more comprehensive if the frailty status assessed by instruments other than FFP were also available. Therefore, this study was conducted to determine the relationship between frailty status, as ascertained according to different criteria, and objectively measured sedentary behavior in older adults.

## Methods

### Study design and setting

This cross-sectional study was conducted at the Department of Geriatrics and Gerontology of a medical center in Taipei, Taiwan. Community-dwelling older adults who were previously enrolled in studies of health examinations (Study 1) or integrated outpatient clinics (Study 2) between September 17, 2020, and October 1, 2021, were recruited for this study to add an accelerometer component to the data obtained. The study protocol was approved by the appropriate research ethics committee and complied with all the ethical rules stated in the Declaration of Helsinki. Written informed consent was obtained from all participants. The study was retrospectively registered on clinical trial platforms (201903110RIND, 202008046RINC).

### Participants

**Fig. 1:**
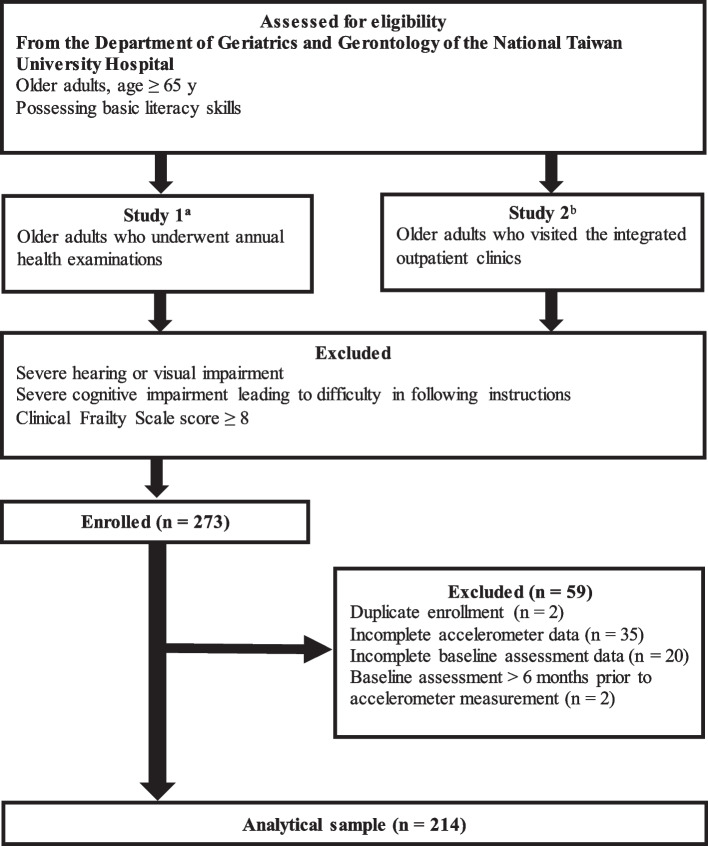
Flowchart of participant recruitment and the data selection process. ^a^ Frailty Risk Assessment and Management in Community-dwelling Elderly. ^b^ Prognostic Implications of Common Geriatric Syndromes on Elderly Outpatients and Hospitalized Patients

Older adults aged 65 y or more with basic literacy skills were eligible for study participation. The inclusion criteria of Study 1 were (1) participation in an annual geriatric health examination and (2) ability to walk independently or with a walker for > 10 m. For Study 2, the inclusion criteria were an outpatient visit to an integrated geriatric clinic by patients having at least one of the following conditions: (1) fall within 1 y, (2) functional decline within 1 y, (3) body weight loss of 5% in 1 month or 10% in 6 months, (4) polypharmacy (≥ 5 medications), (5) urinary incontinence, or (6) osteoporosis. The exclusion criteria were: (1) severe hearing or visual impairment, (2) severe cognitive impairment that could lead to difficulty in following instructions, and (3) a CFS score ≥ 8. Initially, 273 field data points were collected during the study period. We further excluded duplicate data from the same participant (*n* = 2), incomplete accelerometer data (*n* = 35), incomplete baseline assessment data (*n* = 20), and baseline assessment data that were obtained more than 6 months before the accelerometer measurement (*n* = 2). Thus, data from 214 participants were included in the analysis (Fig. 1).

### Measurement of frailty

We adopted the modified Fried Frailty Phenotype (mFFP) with five components: weight loss (> 3 kg in the last year) [30], exhaustion (self-reported fatigue for at least 3 days during the last week) [31], weakness (handgrip strength < 28 kg in men or < 18 kg in women) [32], slowness (6-m walking speed < 1 m/s) [32], and low physical activity (not being physically active beyond walking around during activities of daily living) [33]. Participants without any, one or two, and three or more mFFP components were classified as robust, pre-frail, and frail, respectively.

The CFS-C [34], the Chinese translated version of the CFS, is a 9-point scale that assesses specific domains, including function, cognition, and comorbidity, to generate a frailty score that ranges from 1 (very fit) to 9 (terminally ill). The CFS-C demonstrated a significant correlation with other commonly used frailty criteria (Kendall’s tau, 0.46–0.63). We considered CFS-C scores of 1 to 3 as robust, 4 as pre-frail, and ≥ 5 as frail in this study.

The SOF index comprises the following three criteria: (1) weight loss of more than 3 kg in the past 1 year; (2) difficulty rising from a chair five times without using one’s arms (> 15 s); and (3) self-reported fatigue for at least 3 days during the last week. Pre-frailty was defined as the presence of one component and frailty as ≥ 2 positive criteria. Participants who did not fulfill any of the SOF criteria were considered robust.

The Clinical Frailty-Deficit Count (CF-DC) index [35], which was modified from the FI proposed by Rockwood, comprises 79 risk factors across five domains: lifestyle-related factors (8 items); health status and healthcare-related factors (29 items); nutrition and sarcopenia-related factors (10 items); cognition, mood, and spirituality-related factors (14 items); and functional status-related factors (18 items) (Table 1). Among these factors, 68 variables were ascertained using a self-reported questionnaire, whereas 11 variables comprised measurements of vital signs, oxygen saturation, body composition, hand grip strength, walking speed, Timed Up and Go Test, and five sit-to-stand tests, which were collected using the BabyBot vital data recording system (Netown Corporation, Taipei, Taiwan). The score was calculated by dividing the sum of the deficits, where “with” or “without” the problem described were scored as 1 and 0, respectively, by the total number of parameters that were examined. The final score ranged from 0 to 1. We set 0.25 as the cutoff point for identifying frailty; a score of 0.1–0.25 indicated pre-frailty, whereas a score < 0.1 indicated robustness [35]. Regardless of the criteria adopted, pre-frail or frail participants were categorized as the frail group, whereas the remainder were categorized as the robust group.

**Table 1:** List of variables in the Clinical Frailty-Deficit Count (CF-DC) index

Lifestyle-related variables	Low education level (≤ 6 y), living alone, residence on the 2nd floor and above without elevator, no one can help you when you are in need, lack of social activities, smoking, alcohol intake, no regular exercise
Health status- and healthcare-related variables	Hypertension, diabetes mellitus, hyperlipidemia, cerebrovascular disease, cardiovascular disease, chronic lung disease, liver disease, renal and urinary disorder, cancer, sleep disorder, neurodegenerative disorder, thyroid disease, gastrointestinal disease, hematological disease, bone and joint disease, osteoporosis, spondylopathy, rheumatic disease, gout, visiting ≥ 4 different specialists, polypharmacy (≥ 8 drugs), fall in the past year, not positively accepting medical healthcare, unable to take care of yourself, unable to make medical decisions by yourself, systolic blood pressure > 140 mmHg, diastolic blood pressure > 90 mmHg, pulse rate > 96/min, oxygen saturation < 95%
Nutrition- and sarcopenia-related variables	Have nutritional problems, weight loss > 3 kg within the last year, missing teeth, BMI < 18.5 or > 24 kg/m2, appendicular skeletal muscle index < 7.0 or < 5.7 in men and women, handgrip strength < 28 or < 18 kg in men and women, walking speed < 1 m/s, 5 times sit-to-stand test > 12 s, Time Up and Go Test < 10 s, Time Up and Go Test < 0.3 m/s
Cognition-, mood-, and spirituality-related variables	Poor memory, difficulty in learning, difficulty in communication, poor judgment, forget correct date, feel unhappy, loss of interest, feel that your life is empty, have a fear of death, feel hopeless, cannot accept the physiological change of body due to aging, cannot accept the change of life after retirement, with no religion, did not sign DNR
Functional status-related variables	Hearing impairment, visual impairment, cannot dress by yourself, cannot eat by yourself, cannot get up from bed, stand and sit on the chair by yourself, cannot go to the toilet by yourself, cannot take bath by yourself, cannot buy personal item by yourself, cannot do housework at home, cannot manage money, cannot make phone calls/ ride bus on your own, difficulty in walking ≥ 100 m, difficulty in climbing ≥ 10 stairs, decreased mobility and need the assistance of a cane, poor balance, fatigue, low physical activities, fell everything you did was an effort or could not get going

*BMI* body mass index, *DNR* do not resuscitate

### Measurement of sedentary behavior

The waist-worn accelerometer, ActiGraph wGT3X + (ActiGraph LLC, Pensacola, FL, USA), was used to assess sedentary behaviors. Its sensors detected and measured accelerations caused by body movement in different planes continuously. The collected data from the accelerometer was then downloaded for further processing and analysis. The reliability and validity of accelerometers for measuring sedentary behavior in older adults has been previously reported [36]. We distributed one accelerometer to each participant and asked them to wear the devices for 7 consecutive days, except when bathing, showering, or swimming. During the wearing period, participants were asked to record the time at which they went to bed and got out of bed, and to note any instance of device removal for reasons other than the abovementioned activities. A minimum of 10 h of wearing time per day and at least 5 days of valid data were required for inclusion in the analyses [36]. A threshold of < 100 counts per minute was applied to denote sedentary time [36], whereas ≥ 30 min of consequent sedentary time was defined as one sedentary bout [37]. The sum of the total duration of sedentary bouts per day was measured and defined as the total sedentary time [37]. The data were analyzed using ActiLife Software (v6.13.3 Actigraph Inc., Pensacola, FL).

### Statistical analyses

For the description of baseline characteristics, continuous variables are reported as mean ± standard deviation, and categorical variables as numbers and percentages. Baseline characteristics were compared using analysis of variance to examine the differences in accelerometer measures (total number and total time of sedentary bouts per day). Missing data comprised less than 5% of the total data, and the expectation–maximization algorithm was used to impute incomplete data. Variables with *P* < 0.1 in the bivariate analysis were entered into the stepwise linear regression model to determine the independent effects of the total sedentary time and the number of sedentary bouts per day. The main independent variables of interest were the four frailty indicators that were always included in the models, irrespective of statistical significance at the bivariate level. Statistical analyses were performed using SPSS version 23.0 (SPSS, Inc, Chicago, IL, USA), and significance was set at *P* < 0.05.

## Results

The baseline characteristics of the study population are summarized in Table 2. Among the 214 older participants, 54.2% (*n* = 116) were women, and the mean age was 80.82 ± 7.14 y. The average total sedentary time and number of sedentary bouts were 609.74 ± 79.29 min and 5.51 ± 2.09 times per day, respectively. The prevalence of frailty in older adults as determined by the mFFP, CFS-C, SOF, and CF-DC indices were 67.8%, 28.0%, 50.0%, and 63.6%, respectively.

**Table 2:** Baseline characteristics of the participants

Variables	n (%) or mean ± SD
Sex (female)	116 (54.2%)
Age (y)	80.82 ± 7.14
Height (cm)	158.66 ± 9.10
Weight (kg)	61.24 ± 11.05
BMI (kg/m^2^)	24.27 ± 3.56
Educational level (≤ 6 y)	49 (22.9%)
Living alone	19 (9.0%)
Smoking	15 (7.0%)
Alcohol intake	23 (10.7%)
Regular exercise	75 (35.0%)
Visit to ≥ 4 different specialists	27 (12.7%)
Polypharmacy (≥ 8 drugs)	60 (28.3%)
Number of comorbidities	3.06 ± 2.32
Total sedentary time (min/day)	609.74 ± 79.29
Number of sedentary bouts (times/day)	5.51 ± 2.09
Frailty (positive)	
mFFP	147 (67.8%)
CFS-C	60 (28.0%)
SOF Index	107 (50.0%)
CF-DC Index	136 (63.6%)

*BMI* body mass index, *CF-DC* Clinical Frailty-Deficit Count, *CFS-C* Clinical Frailty Scale, *mFFP* modified Fried Frailty Phenotype, *SD* standard deviation, *SOF* Study of Osteoporotic Fractures

The associations between baseline characteristics and sedentary behavior are presented in Table 3. The significant factors included higher age, smoking, polypharmacy (≥ 8 drugs), and frailty defined by the mFFP and CFS-C for the total sedentary time per day. Furthermore, with regard to the number of sedentary bouts per day, we found that a higher age, male sex, smoking, visits to more than four different specialists, polypharmacy (≥ 8 drugs), and frailty (as defined by all four criteria) were significant factors.

**Table 3:** Association between baseline participant characteristics and accelerometer-measured sedentary behaviors

Variables		Total sedentary time (min/day, mean ± SD)	*P*-value^*^	Number of sedentary bouts (times/day, mean ± SD)	*P*-value^*^
Sex	Male	611.7 ± 72.5	.744	5.90 ± 1.83	.011
	Female	608.1 ± 84.9		5.18 ± 2.24	
Age, y	65–74	583.8 ± 90.2	.031	4.89 ± 1.92	.018
	75–84	615.6 ± 67.4		5.69 ± 1.98	
	≥ 85	625.6 ± 83.4		5.84 ± 2.20	
Education level	≤ 6 y	613.2 ± 91.7	.853	5.73 ± 2.27	.459
	> 6 y	610.8 ± 74.0		5.48 ± 2.02	
BMI	< 18.5	640.6 ± 77.3	.665	5.52 ± 2.54	.060
	18.5–24	608.1 ± 85.1		5.15 ± 1.95	
	≥ 24	608.0 ± 79.3		5.84 ± 2.12	
Living status	Alone	579.1 ± 87.9	.074	5.22 ± 2.17	.478
	With family	614.3 ± 78.5		5.58 ± 2.06	
Smoking	Yes	652.6 ± 109.4	.033	6.77 ± 2.63	.017
	No	607.2 ± 76.2		5.45 ± 2.00	
Alcohol intake	Yes	609.9 ± 96.6	.939	5.75 ± 2.10	.572
	No	610.2 ± 77.6		5.50 ± 2.07	
Regular exercise	Yes	612.6 ± 79.7	.743	5.76 ± 1.97	.235
	No	608.9 ± 79.8		5.41 ± 2.12	
Number of comorbidities	≥ 4	611.9 ± 85.0	.766	5.56 ± 2.13	.783
	< 4	608.5 ± 76.1		5.48 ± 2.07	
Number of specialists visited	≥ 4	625.3 ± 52.4	.289	6.60 ± 1.94	.004
	< 4	608.0 ± 82.5		5.38 ± 2.05	
Polypharmacy	≥ 8 drugs	627.7 ± 73.0	.043	6.62 ± 1.90	< .001
	< 8 drugs	603.3 ± 81.0		5.11 ± 1.98	
mFFP	Frail Robust	618.1 ± 81.6 592.3 ± 71.6	.026	5.96 ± 2.15 4.55 ± 1.57	< .001
CFS-C	Frail Robust	638.4 ± 83.4 598.6 ± 75.0	.001	6.94 ± 2.14 4.94 ± 1.77	< .001
SOF Index	Frail Robust	617.6 ± 86.5 601.9 ± 70.9	.147	5.97 ± 2.16 5.04 ± 1.90	.001
CF-DC Index	Frail Robust	615.4 ± 81.6 599.9 ± 74.6	.170	5.88 ± 2.07 4.85 ± 1.96	< .001

*BMI* body mass index, *CF-DC* Clinical Frailty-Deficit Count, *CFS-C* Clinical Frailty Scale, *mFFP* modified Fried Frailty Phenotype, *SD* standard deviation, *SOF* Study of Osteoporotic Fractures

^*^P-values were based on analysis of variance for all categorical variables

Table 4 shows the associations between the four different indices and two different sedentary behavior outcomes. Linear regression analyses were performed after adjusting for potential confounding variables. When the mFFP definition of frailty was adopted, participants with frailty tended to have a longer total sedentary time (odds ratio [OR] = 30.13, *P* = 0.01) and more frequent sedentary bouts per day (OR = 3.50, *P* < 0.001). Similar results were found when using the CFS-C for frailty diagnosis: the frail older adults had a significantly higher total sedentary time (OR = 39.43, *P* < 0.001) and more frequent sedentary bouts per day (OR = 5.86, *P* < 0.001). As per the SOF index for frailty, a higher odds of increased sedentary bouts was reported for participants in the frail group than those in the robust group (OR = 2.06, *P* = 0.009), but not for the total sedentary time per day. Participants with frailty defined by the CF-DC index were more likely to have more frequent sedentary bouts (OR = 2.03, *P* = 0.016); however, this did not apply to the total sedentary time.

**Table 4:** Adjusted model of the association between four differently frailty indices and sedentary behaviors

Variables	Total sedentary time (min/day)		Number of sedentary bouts (times/day)	
	**OR**	***P*-value**	**OR**	***P*-value**
Frailty by **mFFP**	30.13	.010	3.50	< .001
Sex (male)	N/A*	N/A*	1.90	0.014
Age, y	19.31	.009	1.88	< .001
Polypharmacy (≥ 8 drugs)			3.29	< .001
Frailty by **CFS-C**	39.43	< .001	5.86	< .001
Sex (male)	N/A*	N/A*	2.08	.005
Polypharmacy (≥ 8 drugs)			2.27	.007
Frailty by **SOF** Index	13.29	.225	2.06	.009
Sex (male)	N/A*	N/A*	1.80	.027
Age	6.19	.018	1.79	.001
Smoking	4.37	.041		
Polypharmacy (≥ 8 drugs)			3.95	< .001
Frailty by the **CF-DC** Index	17.22	.134	2.03	.016
Sex (male)	N/A*	N/A*	1.71	.043
Age, y	7.03	.013	1.84	.001
Smoking	4.39	.038		
Polypharmacy (≥ 8 drugs)			3.88	< .001

Blank cells represent non-significant variables in the model

*CF-DC* Clinical Frailty-Deficit Count, *CFS-C* Clinical Frailty Scale, *mFFP* modified Fried Frailty Phenotype, *N/A* not applicable, *OR* odds ratio, *SD* standard deviation, *SOF* Study of Osteoporotic Fractures

^*^N/A indicates that the variable was not included in the model

As shown in Table 4, when frailty was defined by the mFFP, a higher age was positively correlated with both the daily total sedentary time (OR = 19.31, *P* = 0.009) and sedentary bouts (OR = 1.88, *P* < 0.001); male sex and polypharmacy (≥ 8 drugs) were only associated with the number of sedentary bouts (OR = 1.90, *P* = 0.014 and OR = 3.29, *P* < 0.001, respectively). With the CFS-C, male sex and polypharmacy (≥ 8 drugs) were also positively associated with the number of sedentary bouts (OR = 2.08, *P* = 0.005 and OR = 2.27, *P* = 0.007, respectively). When adopting the SOF index, a higher risk of longer sedentary time was found in participants who were older (OR = 6.19, *P* = 0.018) and in smokers (OR = 4.37, *P* = 0.041). With regard to daily sedentary bouts, a positive correlation was observed with the male sex (OR = 1.80, *P* = 0.027), higher age (OR = 1.79, *P* = 0.001), and polypharmacy (OR = 3.95, *P* < 0.001). When using the CF-DC, the significant relationship observed between the variables and the two sedentary behaviors was similar to that observed with the SOF index.

## Discussion

The main finding of this study was that, regardless of the frailty index used, a positive association with the frequency of sedentary bouts was detected in community-dwelling older adults. However, frailty significantly correlated with the daily total sedentary time only when the mFFP and CFS-C scales were used.

According to two analyses defined by the Frailty Trait Scale and 46-item FI, frailty is associated with a higher number of sedentary bouts [26, 29]. The results of our study seem consistent with previous findings. Moreover, our study showed that all four frailty indicators were associated with sedentary bouts. One possible mechanism that explains this association is that frail individuals tend to have poor endurance and easy fatigability and may need to take more breaks during physical activity, which lead to more sedentary bouts per day.

Similar to the results of most studies that used the FFP, our results revealed a positive correlation between frailty and total sedentary time [19, 38–40]. Only one study that used FFP showed no significant correlation for the evaluated variables [28]. Studies that used the FI to assess frailty demonstrated a strong association between frailty and longer sedentary time [20, 22], whereas our study did not detect this association. No study in the existing literature has adopted the CFS-C scale or SOF index to investigate the relationship between frailty and sedentary behavior. Further studies are needed to explore the inconsistent findings between different frailty assessments and the total sedentary time.

In this study, the total time spent in sedentary behavior among older adults was 10.2 h per day. A systematic review that included studies from 10 countries reported that older adults had an average of 9.4 h per day of objectively measured sedentary behavior [15]. The longer sedentary time in our study could be explained by the higher mean age (80.8 y) of the participants compared with that observed in the review (72.2 y).

The characteristics of sedentary older people have been previously described, and include higher age [41, 42], abnormal body mass index [42–45], and smoking [41, 44]. In our study, we found a positive relationship between a higher age and sedentary behavior outcomes under three different definitions of frailty (mFFP, SOF Index, and CF-DC Index). Participants who smoked were inclined to have a longer total sedentary time in a day, based on the SOF and CF-DC indices. Studies have demonstrated that smoking is strongly correlated with physical inactivity, and current smokers tend to exercise less than non-smokers [46].

Among the adjusted models, only male sex and polypharmacy remained significantly associated with an increased frequency of sedentary bouts per day after adjusting for the four different criteria for frailty. Nevertheless, the correlations between sex, polypharmacy, and sedentary behavior are conflicting. Several studies have indicated that sedentary behavior is more prevalent in men [41, 44, 47, 48], whereas others have suggested that women are more likely to be physically inactive [43, 45]. Satariano et al. considered that different family priorities and responsibilities may explain why men tend to be more sedentary than women. Women are inclined to spend more time on caregiving then men, resulting in less sedentary time [49]. One study showed that the number of medications taken was positively associated with sedentary behavior [50], and another study found no significant association between polypharmacy (≥ 5 drugs) and sedentary behavior [51].

To our knowledge, this is the first study to use four commonly used criteria for frailty in analyses of the association of frailty with sedentary behavior. However, this study had some limitations. First, the causal relationship between frailty and sedentary behavior could not be established because of the cross-sectional study design. Second, the participants were mostly older people who were living in urban areas (Taipei), and data from rural areas were not included. Therefore, the results do not represent the overall conditions of the Taiwanese population. Third, the validity and reliability of the CF-DC index have only been verified in domestic research. Fourth, there were differences in the definitions of sedentary time. To achieve a meaningful quantum of sedentary time, we set a threshold of 30 min as the total sedentary time, which was not specifically regulated in other studies. Fifth, the classification of frailty differed from that used in other research, which mostly classified the frailty status into three stages: robust, pre-frail, and frail. In contrast, we included pre-frail and frail participants in the frail group.

## Conclusions

In conclusion, frailty was positively associated with the number of sedentary bouts per day in older adults for all four of the frailty indices that were used for the assessment. However, when the total sedentary time was used as an outcome, a significant association was found only with the use of the mFFP and CFS-C scales. Further research may target decreasing the sedentary bouts in older adults as a strategy to improve frailty.

## Data Availability

The datasets used and/or analysed during the current study are available from the corresponding author on reasonable request.

## Abbreviations

CFS-C: Clinical Frailty Scale in Chinese Translation
CF-DC: Clinical Frailty-Deficit Count
CFS: Clinical Frailty Scale
FFP: Fried Frailty Phenotype
FI: Frailty index
mFFP: Modified Fried Frailty Phenotype
OR: Odds ratio
SOF: Study of Osteoporotic Fractures
